# Clinical Audit : Supporting Post Detox Abstinence: Discussion of Relapse Prevention Medications by Community Addiction Services Prior to Referral for Inpatient Detoxification

**DOI:** 10.1192/bjo.2025.10700

**Published:** 2025-06-20

**Authors:** Farheen Zahra, John Barker, Paul Briley

**Affiliations:** 1 Nottinghamshire Healthcare NHS Foundation Trust, Nottingham, United Kingdom; 2 University of Nottingham, Nottingham, United Kingdom

## Abstract

**Aims:** Without a plan to support ongoing abstinence, detoxification (“detox”) could increase, rather than reduce, risks to a patient. Before referring for inpatient detox from alcohol or opioids, community teams are expected to discuss relapse prevention medications (RPMs) with patients, as part of their wider support plan.

This clinical audit examined whether RPMs were mentioned in referrals by community teams to our inpatient detox unit.

**Methods:** We examined referrals for patients admitted to The Level Nottingham inpatient detox unit between 1 January and 31 August 2024. Of a total of 215 patients that completed opioid or alcohol detox, a random sample of 50 were selected, stratified according to referring team. Referral forms and running notes were used to assess compliance with the following criteria:

1. Referring teams mention RPMs (whether to be considered or not considered).

2. Referring teams provide blood test investigations.

There was no previous literature or audit to specify a standard, so, given the importance of the issues under consideration, this was set as 100% for each criterion. We also extracted: whether patients were planned to go to residential rehabilitation after detox, and, where relevant, which RPMs were mentioned and time from blood test results to referral and to admission.

**Results:** 68% of referrals were for alcohol, and 24% for opioid, detoxification (2% were for alcohol and opioid, and 6% for other substances).

40% of referrals for alcohol, and 77% of referrals for opioid, detoxification did not mention RPMs.

29% of referrals for alcohol, and 31% of referrals for opioid, detoxification did not mention RPMs and were not planned to go to residential rehabilitation (considered as some of these settings do not accept patients on RPMs, focusing solely on psychosocial support).

48% of referrals for any detoxification did not have blood test results available. Where blood test results were available, median time from test results was 22 days to referral and 85 days to admission.

**Conclusion:** During the study period, an estimated one-third of referrals for alcohol or opioid detoxification did not mention RPMs (and were not going to residential rehabilitation post inpatient stay).

Approximately half of admissions did not have blood test results available.

The above is likely to delay the prescription of RPMs, and potentially increase the risk of relapse post-detoxification.

Recommendations to increase performance include discussions with referrers, changes to the referral form, and changes to referral screening.

